# Long‐term daily oral administration of intestinal permeation enhancers is safe and effective in mice

**DOI:** 10.1002/btm2.10342

**Published:** 2022-05-31

**Authors:** Katherine C. Fein, John P. Gleeson, Kyle Cochran, Nicholas G. Lamson, Rose Doerfler, Jilian R. Melamed, Kathryn A. Whitehead

**Affiliations:** ^1^ Department of Chemical Engineering Carnegie Mellon University Pittsburgh Pennsylvania USA; ^2^ Department of Biomedical Engineering Carnegie Mellon University Pittsburgh Pennsylvania USA

**Keywords:** intestinal epithelium, oral drug delivery, permeation enhancer

## Abstract

Although protein drugs are powerful biologic therapeutics, they cannot be delivered orally because their large size and hydrophilicity limit their absorption across the intestinal epithelium. One potential solution is the incorporation of permeation enhancers into oral protein formulations; however, few have advanced clinically due to toxicity concerns surrounding chronic use. To better understand these concerns, we conducted a 30‐day longitudinal study of daily oral permeation enhancer use in mice and resultant effects on intestinal health. Specifically, we investigated three permeation enhancers: sodium caprate (C_10_), an industry standard, as well as 1‐phenylpiperazine (PPZ) and sodium deoxycholate (SDC). Over 30 days of treatment, all mice gained weight, and none required removal from the study due to poor health. Furthermore, intestinal permeability did not increase following chronic use. We also quantified the gene expression of four tight junction proteins (claudin 2, claudin 3, ZO‐1, and JAM‐A). Significant differences in gene expression between untreated and permeation enhancer‐treated mice were found, but these varied between treatment groups, with most differences resolving after a 1‐week washout period. Immunofluorescence microscopy revealed no observable differences in protein localization or villus architecture between treated and untreated mice. Overall, PPZ and SDC performed comparably to C_10_, one of the most clinically advanced enhancers, and results suggest that the chronic use of some permeation enhancers may be therapeutically viable from a safety standpoint.

## 1 INTRODUCTION

Oral delivery is the patient‐preferred route of drug administration because it is convenient and pain‐free; unfortunately, it is currently not possible for biologics such as peptide and protein drugs.^1^ This is because macromolecule therapeutics are degraded in the stomach and are not absorbed across the small intestine and into the bloodstream. While the former challenge can be addressed with protective enteric coatings, the latter has prevented the oral delivery of most molecules larger than ~500 Da.^2^, ^3^ Many of the most widely used drugs on the market, including insulin (5.7 kDa) and adalimumab (HUMIRA®, 144 kDa), are macromolecule drugs offered only via subcutaneous or intravenous injection.^4^ There is a strong incentive to enhance the intestinal absorption of these drugs so they can be delivered orally, which will improve patient compliance and quality of life. The relevance of oral macromolecular delivery is expected to increase as more protein drugs are approved and other biologics such as nucleic acid‐based therapeutics are developed.

The most common strategy to facilitate the intestinal absorption of orally delivered macromolecules is the use of permeation enhancers.^5^, ^6^ These molecules affect the intestinal epithelium, which is a single layer of columnar epithelial cells, to increase its permeability. Permeation enhancers typically act through one of two mechanisms. Some enhancers increase paracellular permeability, or the transport between cells, through molecular manipulation of the protein complexes that connect the epithelial cells to one another.^5^, ^7^ These protein complexes, called tight junctions, maintain the polarity and barrier function of the epithelium.^8^, ^9^ Alternatively, some enhancers act transcellularly, meaning that they increase transport through intestinal epithelial cells. This can occur through upregulation of receptor‐specific transport processes or by disrupting the lipid membrane of the epithelial cells via fluidization.^7^, ^10^ A number of oral biopharmaceutical formulations containing permeation enhancers are in clinical trials, and the first oral peptide formulation including a permeation enhancer was approved by the FDA in 2019.^11^

The permeation enhancers that have made clinical progress have often been limited to generally recognized as safe (GRAS) substances and food additives. For example, sodium caprate (C_10_) is a native component of dairy products and a common food additive. C_10_ has been the subject of numerous cell culture, preclinical, and clinical studies, making it one of the most widely studied permeation enhancers, and its safety in humans is well established.^12^, ^13^, ^14^, ^15^, ^16^ However, C_10_ is an anomaly, and the majority of permeation enhancers have not been examined for their effects following repeat dosing in animals or in humans.

Because only a handful of permeation enhancers have translated into the clinic, there is pessimism surrounding their utility. The primary concern is that chronic absorption enhancer use will cause toxicity, either due to cumulative epithelial damage or the unwanted passage of toxic substances into systemic circulation. However, there are few published studies in animals or humans that validate this concern in a repeat dosing scenario.^17^ Most reports on permeation enhancers present efficacy and toxicity data from cell culture experiments or from animal experiments that include only a single dose.^16^, ^18^, ^19^ As such, it is not understood how enhancer chemistry and mechanism of action affect patient health following chronic dosing, and this knowledge gap hinders the rational design of next‐generation permeation enhancers.

To address this knowledge gap, we evaluated the effects of three oral permeation enhancers in mice that were delivered once daily for 30 days. Here, we show that chronic enhancer dosing did not produce negative health outcomes and that the modest changes observed for some endpoints (e.g., gene expression) resolved after a 1‐week washout period. Together, these data suggest that chronic dosing of a broader range of permeation enhancers may be a clinically viable strategy for oral macromolecule and protein delivery.

## 2 METHODS

### 2.1 Materials

Phosphate buffered saline (PBS), AlexaFluor‐antibody conjugates, DAPI, Fluoromount‐G™ and primers were purchased from Thermo Fisher (Waltham, MA). Zonulin ELISA kit was purchased from Abcam (Cambridge, UK). Hemoccult Guaiac Fecal Occult Blood Test slides and serum collection tubes were obtained from VWR (Radnor, PA). FITC‐Dextrans, PPZ, SDC, and C_10_ were purchased from Millipore Sigma (Burlington, MA). Plastic oral gavage needles were sourced from Instech Laboratories, Inc. (Plymouth Meeting, PA).

### 2.2 Animal care and use

Animal protocols were approved by the Institutional Care and Use Committee at Carnegie Mellon University (Pittsburgh, PA), and all experiments were performed in accordance with protocol PROTO201600017 as well as all institutional, local, and federal regulations. Female C57/BL6 mice aged 10 weeks were purchased from Charles River Laboratories and acclimated to facility conditions for 4 weeks before the study began. Mice were housed in standard cages with a 12‐h light/dark cycle and free access to water and food.

### 2.3 One day time point permeability study

Mice were fasted for 12 h prior to the start of the experiment in cages with fasting grates and no food or bedding but free access to water. Negative control mice were orally gavaged with 600 mg/kg 4 kDa FITC‐dextran (FD4) dissolved in PBS. Treated mice received either 60 mg/kg 1‐phenylpiperazine (PPZ), 200 mg/kg sodium deoxycholate (SDC), or 390 mg/kg sodium caprate (C_10_) dissolved in PBS in addition to 600 mg/kg FD4. All solutions were dosed at 10 μl/g of body weight. Blood samples were taken from the submandibular vein at 0, 0.5, 1, 1.5, and 3 h postgavage. Blood was collected in serum tubes (VWR, Radnor, PA) and centrifuged at 15,000 ×g for 10 min to isolate the serum. 10 μl of serum was diluted 1:10 with PBS in black 96‐well plates, and fluorescence was measured on a BioTEK Synergy H1 plate reader at 490 nm excitation and 520 nm emission wavelengths. A calibration curve of FD4 was prepared for each experiment to calculate the concentration of FD4 in the blood. A blank fluorescence value of serum from an untreated mouse (not one of the subjects of this study) was subtracted from each measurement to account for autofluorescence of biological materials at these wavelengths. After 3 h, mice were sacrificed, the small intestine and colon were dissected, and tissue samples were collected for RNA extraction (see section on qRT‐PCR).

### 2.4 Baseline permeability measurement

One week before the study began, baseline intestinal permeability was measured as follows. Mice were fasted for 12 h prior in cages with fasting grates and no food or bedding but free access to water. Then, mice were orally gavaged with 600 mg/kg FD4. After 3 h, blood was taken from the submandibular vein and mice were returned to standard cages and fasting was ended. FD4 concentrations in the blood were determined as described above.

### 2.5 Safety study design

The experiment began when mice were 14 weeks old. Mice were weighed daily, and their condition was monitored. Conditions requiring sacrifice for humane considerations were defined as mice losing 20% or more of their body weight. Mice were randomly assigned to treatment groups prior to the start of the study.

On Day 1 of the study, mice were weighed and received 600 mg/kg FD4 dissolved in PBS with no treatment, 60 mg/kg PPZ, 200 mg/kg SDC, or 390 mg/kg C_10_ by oral gavage. After 3 h, blood was taken from the submandibular vein, and mice were returned to standard cages and fasting was ended. Blood concentrations of FD4 were evaluated as described above. This procedure to measure intestinal permeability was repeated weekly for 4 weeks, on Days 8, 15, 22, and 30.

On all other days (2–29), mice were weighed and received PBS, 60 mg/kg PPZ, 200 mg/kg SDC, or 390 mg/kg C_10_ via oral gavage. On Day 30, the protocol for treatment and intestinal permeability measurement was carried out as previously done, and then the treatment groups were randomly subdivided into two groups each. One subgroup (chronic exposure group) was sacrificed on Day 30. Blood was collected by cardiac puncture, the small intestine and colon were removed, and sections were collected for RNA extraction and histology.

The other subgroup (washout group) was returned to normal cages for a one‐week recovery period during which mice received no treatment. Mice were weighed daily. On Day 37, mice were weighed and orally gavaged with 600 mg/kg FD4 in PBS with no added permeation enhancers. After 3 h, blood was taken to assess FD4 serum concentrations as described above. Then the mice were sacrificed, and blood and tissue samples were taken as described above.

### 2.6 Fecal scoring

Mouse feces were collected on each of the permeability measurement days and assigned a score from 0 to 3 based on solidity, presence of mucus, and presence of blood as determined using Hemoccult Guaiac Fecal Occult Blood Test slides.

### 2.7 Histology

After dissection, small intestine and colon samples were immediately put into 4% formaldehyde for 24 h. Then samples were rinsed with PBS, transferred to 30% sucrose, and stored at 4°C. Samples were embedded in Sakura Tissue‐Tek Optimal Cutting Temperature Compound (OCT; VWR) and stored at −80°C. Samples were sectioned to 10 μm thickness on a Shandon Cryotome® (Thermo Fisher) and then stained as follows.

### 2.8 Immunofluorescence and confocal microscopy

Samples were rinsed in PBS to remove the residual OCT and blocked with 10% (w/v) bovine serum albumin in 0.1% (v/v) TritonX‐100 for 2 h at room temperature. Samples for tight junction staining were incubated with a primary antibody against Claudin 3 overnight at 4°C. Then the samples were rinsed and incubated with anti‐rabbit Alexa‐Fluor 488 for secondary staining of Claudin 3 and anti‐ZO1 AlexaFluor 594 for 2 h. The samples were rinsed again, and Hoechst was added for 25 min to stain the nuclei. Samples were rinsed a final time, mounted in Fluoromount Gold, and coverslips were added. Samples for actin staining were blocked in 10% BSA in TritonX overnight at 4°C. Then the samples were rinsed, and Phalloidin‐Alexa‐Fluor 594 was added for 45 min. Samples were rinsed again and then incubated with Hoechst for 25 min. Samples were rinsed a final time, mounted in Fluoromount Gold, and coverslips were added. Confocal microscopy was performed using a Zeiss LSM 700 using Zen 2012 software (Carl Zeiss, Inc.) with 405, 488, and 555 nm filters.

### 2.9 RNA extraction and real‐time quantitative polymerase chain reaction

After dissection, tissue samples from the small intestine and colon were immediately placed into RNA*later* (Thermo Fisher) and stored at −20°C until they were processed. Tissue samples were placed in 250 μl Trizol reagent (Thermo Fisher) and homogenized with the BeadBug Microtube Homogenizer. 150 μl chloroform was added, and samples were centrifuged for 15 min at 12,000 rpm. The aqueous layer was removed to a fresh 1.5 ml RNAse‐free tube, and an equal volume of ethanol was added. The sample was briefly mixed and then transferred to a spin column from the QIAGEN RNeasy Mini Kit. Further washing steps were performed according to the manufacturer's instructions and using buffers provided in the kit. cDNA was synthesized from 2000 ng of each RNA sample using Applied Biosystems High‐Capacity cDNA Reverse Transcription Kit. Real‐time quantitative polymerase chain reaction (qRT‐PCR) was performed on a Viia 7 Real‐Time PCR System (Applied Biosystems) using SYBR Select Master Mix (Applied Biosystems). Primer sequences can be found in Table 1.

**TABLE 1:** PCR primer sequences

Gene	Forward	Reverse
*β‐Actin*	CACTGTCGAGTCGCGTCC	TCATCCATGGCGAACTGGTG
*Claudin 2*	GAAAGGACGGCTCCGTTTTC	CAGTGTCTCTGGCAAGCTGA
*Claudin 3*	GTACAAGACGAGACGGCCAA	GGGCACCAACGGGTTATAGA
*ZO‐1*	CTCTTCAAAGGGAAAACCCGA	GTACTGTGAGGGCAACGGAG
*JAM‐A*	TCCCGAGAACGAGTCCATCA	GAACTTCCACTCCACTCGGG

### 2.10 Serum zonulin and TNF‐α quantification by ELISA

After the mice were sacrificed, blood was collected by cardiac puncture and centrifuged to isolate the serum. The serum concentrations of zonulin and TNF‐α were measured using enzyme‐linked immunosorbent assay (ELISA) kits purchased from Abcam. Samples were assessed according to the manufacturer's instructions with a 1:1000 dilution factor for zonulin and a 1:100 dilution factor for TNF‐α.

### 2.11 Statistics

For the time point study, group size was *n* = 6. For the longitudinal experiment, mice were randomly assigned to treatment groups such that the initial group size was *n* = 12. All data are presented as the mean with error bars representing the standard error of mean (SEM). Significance was determined by two‐tailed, unpaired Student's t‐tests performed in GraphPad Prism 8.

## 3 RESULTS

The goal of this study was to determine whether chronic administration of intestinal permeation enhancers would cause toxicity. We chose to examine two enhancers known to increase intestinal permeability through distinct mechanisms of action. One of the chosen permeation enhancers is phenylpiperazine (PPZ), which we and others have found to improve paracellular permeation by tight junction rearrangement using in vitro (Caco‐2 monolayers) and ex vivo (rodent intestinal tissue in Ussing chambers) models.^20^, ^21^, ^22^ We also used sodium deoxycholate (SDC), which is a bile salt that enhances permeability via membrane fluidization and tight junction rearrangement as determined using in vitro and ex vivo models.^23^, ^24^ Then, we compared them to sodium caprate (C_10_), as its nontoxicity and efficacy are extensively characterized in literature.^14^, ^15^

The enhancer concentrations used in this study were chosen based on literature data and our previous work.^25^ For PPZ, the oral LD_50_ in rats is published as 210 mg/kg, and we decided upon 60 mg/kg, which increases FD4 absorption while staying below potentially harmful doses. SDC is an endogenous bile salt secreted by the gallbladder to aid the digestion of fat in the intestine. In humans, one study measured fed‐state concentrations between 0.74 and 86.14 mM.^26^ For this study, we chose a dose of 200 mg/kg (equivalent to ~48 mM, assuming dilution in the volume of the intestine). According to the MSDS, the oral LD_50_ for SDC in mice is over 1 g/kg, which is fivefold higher than the dose used in this experiment. The concentration for C_10_ (390 mg/kg, corresponding to 200 mM) was chosen based on an extensive compilation of in vivo studies reviewed by Maher et al.^15^ Our chosen concentration falls in the range of previously used doses and is well below the oral LD50 published for rats, which is 3.7 g/kg.^15^

As is common in the oral protein delivery field, we chose to use FD4 as a model macromolecular drug for this study. FITC‐dextrans are excellent model drugs for oral delivery research not only because they are available in a wide range of molecular weights and relatively inexpensive compared to true protein drugs, but also because they are nondigestible.^27^ This means that they can be dosed orally and assumed to pass through the acidic and denaturing conditions of the stomach without being degraded.

### 3.1 All enhancers increased the intestinal permeation of 4 kDa FITC‐dextran

**FIGURE 1:**
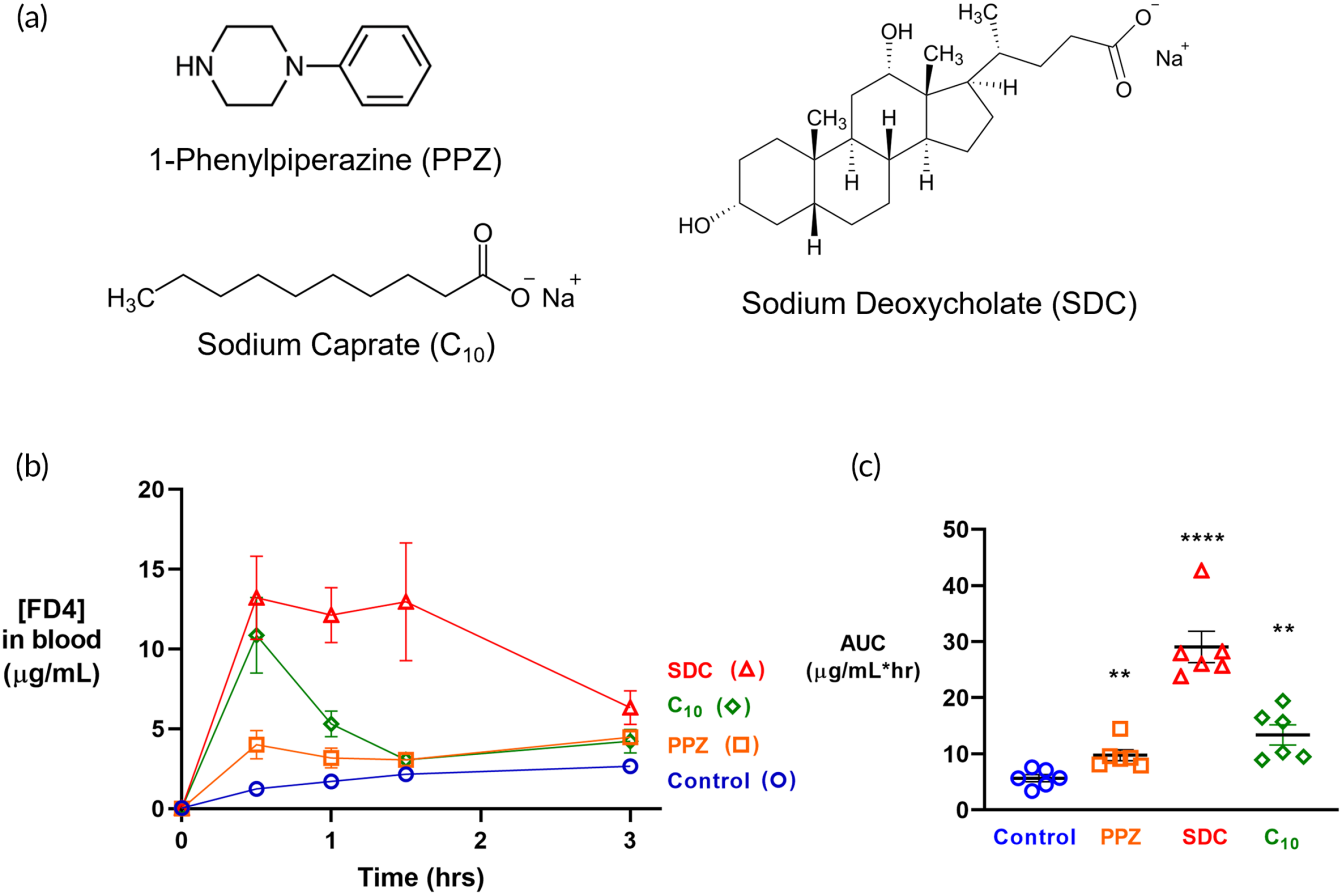
The permeation enhancers 1‐phenylpiperazine (PPZ), sodium deoxycholate (SDC), and sodium caprate (C_10_) increased oral macromolecular absorption. FITC‐dextran 4 kDa (FD4) was co‐delivered to mice with either PBS (Control), PPZ, SDC, or C_10_ by oral gavage. Chemical structures are shown in (a). (b) The concentration profiles varied between the enhancers, with C_10_ and SDC producing rapid increases in blood concentration that dropped by hour 2 and PPZ producing a steady increase in blood concentration over 3 h. (c) All enhancers increased the area under the curve (AUC) of FD4 compared to control over 3 h. *n* = 6, error bars represent SEM, **p < 0.01, ****p < 0.0001 compared to untreated control by unpaired, two‐tailed Student's t‐test.

Before beginning the month‐long safety study, a 1‐day experiment confirmed that our chosen permeation enhancers, PPZ, SDC, and C_10_ significantly increased permeability and established the kinetics of each enhancer's activity. To assess the efficacy of the selected permeation enhancers, we measured the oral absorption of 4 kDa FITC‐dextran (FD4) co‐delivered in solution with PBS (untreated control), 60 mg/kg PPZ, 200 mg/kg SDC, or 390 mg/kg C_10_. Blood samples were taken 30–180 min after administration to determine FD4 blood serum concentrations (Figure 1b). When no permeation enhancer was administered, FD4 serum concentrations were low, and the resultant area under the curve (AUC) was 5.67 ± 0.64 μg/ml*h (Figure 1c). In contrast, mice treated with an enhancer absorbed significantly more FD4, with each enhancer producing unique concentration profiles over time.

SDC caused the largest increase in permeation, with FD4 blood concentration peaking at 30 min and remaining elevated compared to the untreated control until 3 h after administration. C_10_ also rapidly increased FD4 blood concentration, which reached its maximum by 30 min. This increased permeation caused by C_10_ did not persist as long as that caused by SDC. PPZ also enhanced permeation with statistical significance, although to a lesser degree than C_10_ and SDC. All permeation enhancers caused statistically significant increases in the AUC compared to the untreated control, producing fold increases of 1.7, 5.0, and 2.3 for PPZ, SDC, and C_10_, respectively. Because the enhancer doses in this experiment increased the absorption of FD4 after oral gavage, we used them for the remainder of the study.

### 3.2 A single dose of permeation enhancers alters mRNA expression of tight junction proteins

To assess whether one dose of a permeation enhancer affects the structure of the intestine on a molecular level, we measured the mRNA expression of four tight junction proteins in the small intestine and colon 3 h following treatment. We chose to evaluate gene expression after 3 h even though permeability effects are seen within 30 min because collecting tissue for mRNA analysis requires sacrificing the animal, whereas blood collection for permeability analysis can be done repeatedly over a time course experiment. We used qRT‐PCR to measure the relative gene expression of *claudin 2*, a pore‐forming tight junction protein, *claudin 3*, a barrier‐forming tight junction protein, *junctional adhesion molecule‐A (JAM‐A)*, and *zonula occludens‐1 (ZO‐1)*. JAM‐A is critical to the regulation of permeability and inflammation in the intestinal epithelium, and ZO‐1 has contact with most other tight junction proteins and the actin cytoskeleton.^28^, ^29^

**FIGURE 2:**
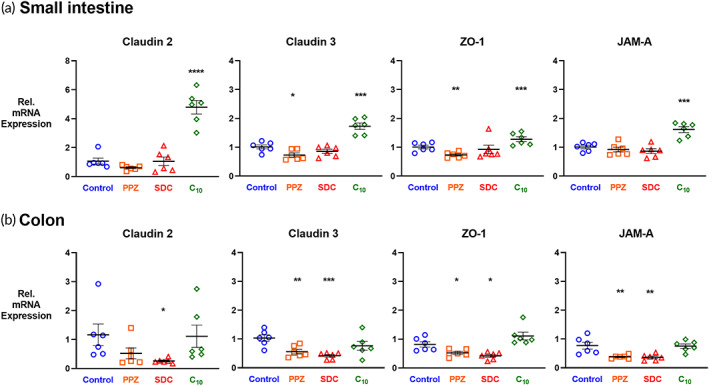
The permeation enhancers PPZ, SDC, and C_10_ affected gene expression of tight junction proteins. Mice received a single dose of PBS (Control) or one of the three enhancers examined in this study. After 3 h, small intestine and colon tissue samples were collected, and mRNA expression was determined by qRT‐PCR for four tight junction proteins: pore‐forming *claudin 2*, barrier‐forming *claudin 3*, *ZO‐1*, and *JAM‐A*. (a) In the small intestine, PPZ and C_10_ induced significant gene expression changes compared to the control. (b) In the colon, PPZ and SDC altered gene expression compared to the control. *n* = 6, error bars represent SEM, *p < 0.05, **p < 0.01, ***p < 0.001, ****p < 0.0001 by unpaired, two‐tailed Student's t‐test.

Figure 2 illustrates that just one dose of C_10_ caused expression increases for all four of the tight junction proteins in the small intestine. One dose of PPZ caused significant decreases to *claudin 3* and *ZO‐1* in the small intestine. In the colon, a single dose of PPZ or SDC affected expression of each of the tight junction proteins.

### 3.3 Repeat dosing of permeation enhancers does not permanently impair intestinal barrier function

To evaluate the effect of long‐term oral administration of permeation enhancers on intestinal barrier function, we designed a month‐long experiment during which mice received daily oral gavages of PBS, PPZ, SDC, or C_10_ at the concentrations used previously. One week prior to the experiment, each animal's baseline intestinal permeability to FD4 was measured following oral gavage, with serum concentrations measured 3 h after administration. On Day 1 of the study, mice received FD4 in solution with one of the permeation enhancers or PBS for the untreated control, and permeability was again measured 3 h after administration. On each of the next 30 days, mice were orally gavaged with permeation enhancer treatments. For ethical reasons regarding the frequency of blood draws, permeability was measured only once per week. On Day 30, half of the mice in each group were sacrificed, and tissues were collected for further analysis. The remaining half of the mice underwent a one‐week washout period (no treatment), after which intestinal permeability to FD4 was assessed a final time.

**FIGURE 3:**
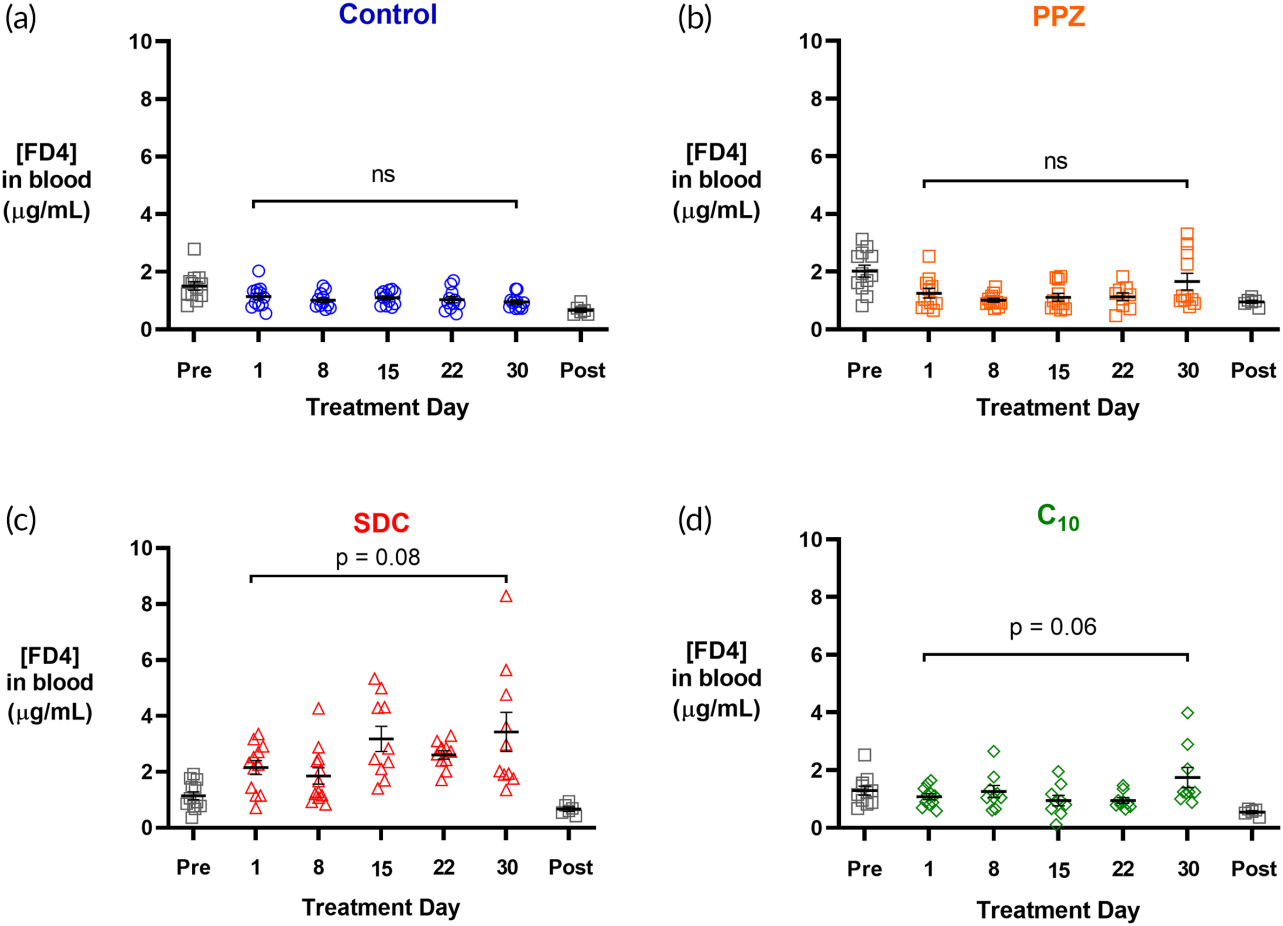
Enhancers did not permanently increase intestinal permeability after 4 weeks of daily oral administration. Baseline untreated intestinal permeability was measured 1 week before treatment began (gray squares‐Pre). Mice were dosed with (a) PBS, (b) PPZ, (c) SDC, or (d) C_10_ every day for 30 days, and the concentration of FD4 in the blood was measured five times throughout the 30‐day period and again after a 1‐week washout period (gray squares‐Post). Over the course of treatment, SDC and C_10_ caused slight increases in permeability that were not significant, while the Control and PPZ groups had no difference between the permeability on Days 1 and 30. The observed increases in FD4 permeability for the SDC and C_10_ groups were no longer present after the washout period. *n* = 6–12, error bars represent SEM. No statistical differences were found between any groups, with significance defined as *p* < 0.05 by unpaired, two‐tailed Student's t‐tests.

The critical endpoint in this study was intestinal permeability, for which increasing values would indicate cumulative damage to intestinal barrier function. This was assessed by measuring FD4 serum concentrations throughout the entire experiment (Figure 3). All values shown as light gray squares are measurements made on days where no permeation enhancer treatment was given. All values shown as colored shapes are measurements made on days where mice were treated with their respective permeation enhancer.

The key comparisons included permeability differences between Days 1 and 30 and between the pretreatment and posttreatment period. In all groups, there were no differences between FD4 concentrations on Days 1 and 30, meaning that the effect of each permeation enhancer was consistent throughout 1 month of daily dosing. While not statistically significant, SDC and C_10_ increased FD4 concentrations by the final day of treatment. However, after the week‐long washout period, no differences from the pretreatment intestinal permeability were observed. These findings do not support the common concern that repeated use of permeation enhancers may cause long‐term increases in intestinal permeability.

### 3.4 Chronic permeation enhancer exposure does not negatively affect GI health indicators

**FIGURE 4:**
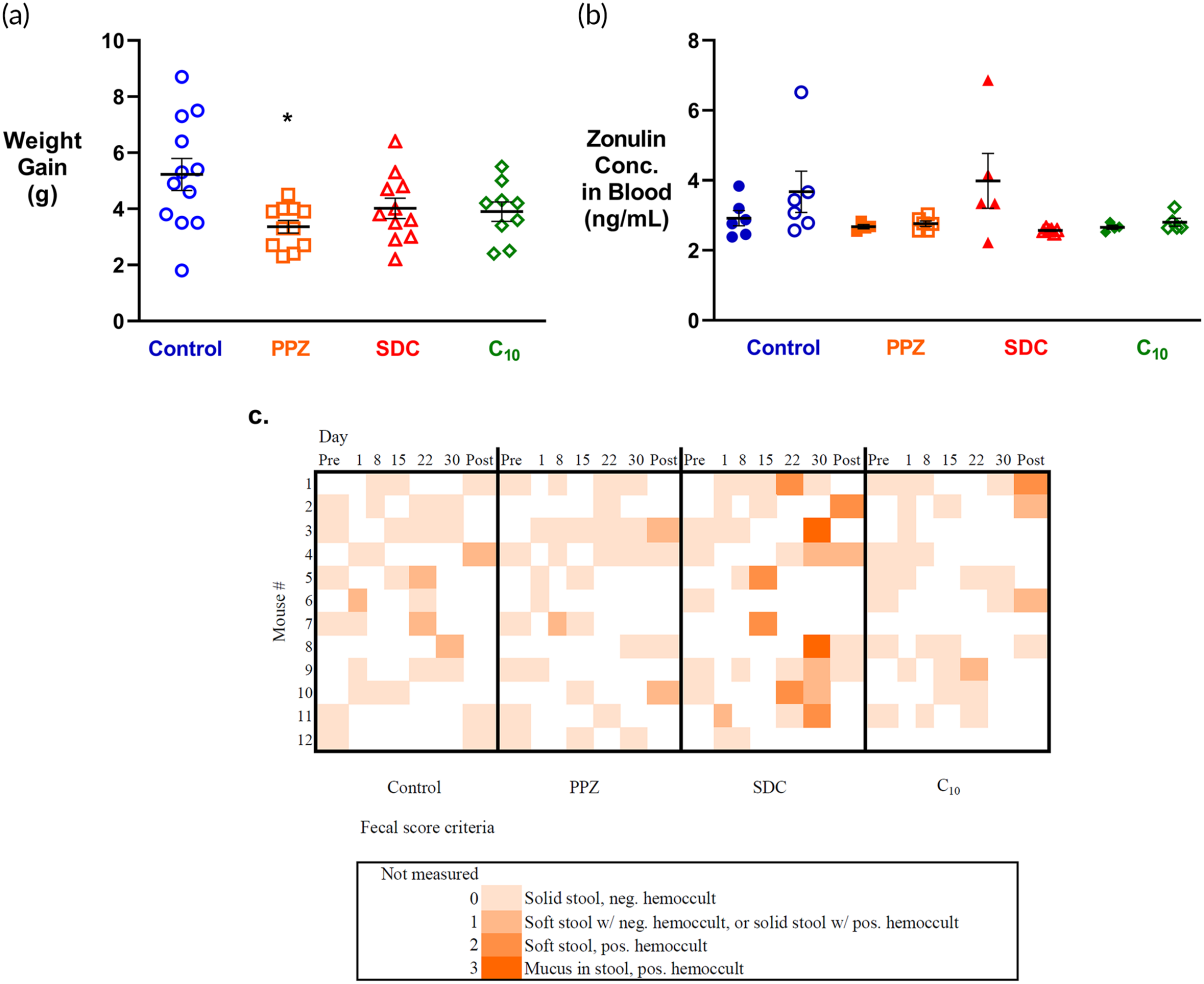
Chronic permeation enhancer exposure does not negatively affect health indicators. (a) Daily permeation enhancer treatment did not cause weight loss. Nonfasted mice were weighed daily during the study. Shown here is the weight gain between 1 week before the study began and treatment day 29. While all groups showed weight gain, weight gains were smaller in the PPZ group compared to the control group. *n* = 9–12, error bars represent SEM, *p < 0.05 by a two‐tailed, unpaired Student's t‐test. (b) Serum zonulin concentrations were not elevated by enhancer treatment. Serum was collected from mice on treatment day 30 (solid symbols) and after a 1‐week washout period (open symbols), and zonulin concentration was measured by ELISA. *n* = 4–6, error bars represent SEM. (c) Only SDC affected the quality of mouse stool. Once per week, mouse stool was collected and assessed for stool solidity, mucus in the stool, and blood in the stool (pos. hemoccult). Only SDC administration caused an increase in fecal score over time with the effect decreasing after a 1‐week washout period.

In addition to intestinal permeability, we measured several other indicators of GI health in mice following a 1‐month exposure to permeation enhancers. Mice were weighed daily, as well as assessed for general behavioral signs of healthy versus stressed states, including barbering, hunching, and fecal abnormalities including mucus in the stool and diarrhea. Regarding weight, mice across all groups gained between 2 and 9 g over the treatment period, with an average weight gain of 4.2 ± 1.5 g (Figure 4a). Only the PPZ group gained significantly less weight (3.4 g) compared to the control group (5.3 g). We believe, however, that the reduced variability in the weights of the PPZ group may confound a conclusion that reduced weight gain was caused by PPZ treatment.

We measured the serum concentrations of the protein zonulin, an increase in which would indicate increased intestinal permeability.^30^, ^31^ These concentrations were assessed by ELISA for mice sacrificed on treatment Day 30 and after the washout period (Figure 4b). None of the treatment groups had significantly different zonulin concentrations compared to the control group. To better understand changes induced by SDC treatment, we also measured TNF‐α concentrations for the control and SDC groups on treatment Day 30. However, no differences were found (data not shown), indicating that SDC did not promote local inflammation mediated by TNF‐α.

We also assessed stool quality, as it worsens when the intestinal epithelium is damaged.^32^ Fecal samples were collected on permeation measurement days and visually inspected for solidity and mucus. Further, blood in the stool was identified using Hemoccult testing. These three measurements were aggregated into a quantitative fecal score between 0 and 3 (Figure 4c). Scores varied from 0 to 1 in the untreated group, suggesting that stool solidity and the presence of blood fluctuate modestly in healthy mice. Fecal scores of PPZ‐ and C_10_‐ treated mice were similar to untreated mice, but scores for SDC‐treated mice increased over time. On the last day of treatment, control, PPZ, SDC, and C_10_ groups had average scores of 0.25, 0, 1.6, and 0, respectively. After the washout period, the average fecal score of the SDC group decreased to 0.75, suggesting that the changes in fecal quality due to SDC treatment were temporary. It is possible that a lower dose of SDC may be a better choice if it does not cause these changes in stool while remaining effective.

No mice were sacrificed over the course of the study for reaching predetermined indicators of poor health. These criteria included loss of ≥20% of their body weight, excessive grooming or barbering, >3 consecutive days of diarrhea or stool containing mucus or blood, and hunching that did not resolve within a few minutes after handling. The only observed instances of barbering were resolved by separating a dominant mouse from its cagemates and housing it separately for the rest of the study. During the study, three mice in the C_10_ group and one in the SDC group were sacrificed due to procedural complications resulting from the oral gavage, and one mouse in the PPZ group was sacrificed due to complications from a blood draw.

### 3.5 Repeated dosing of permeation enhancers affects mRNA expression of tight junction proteins

**FIGURE 5:**
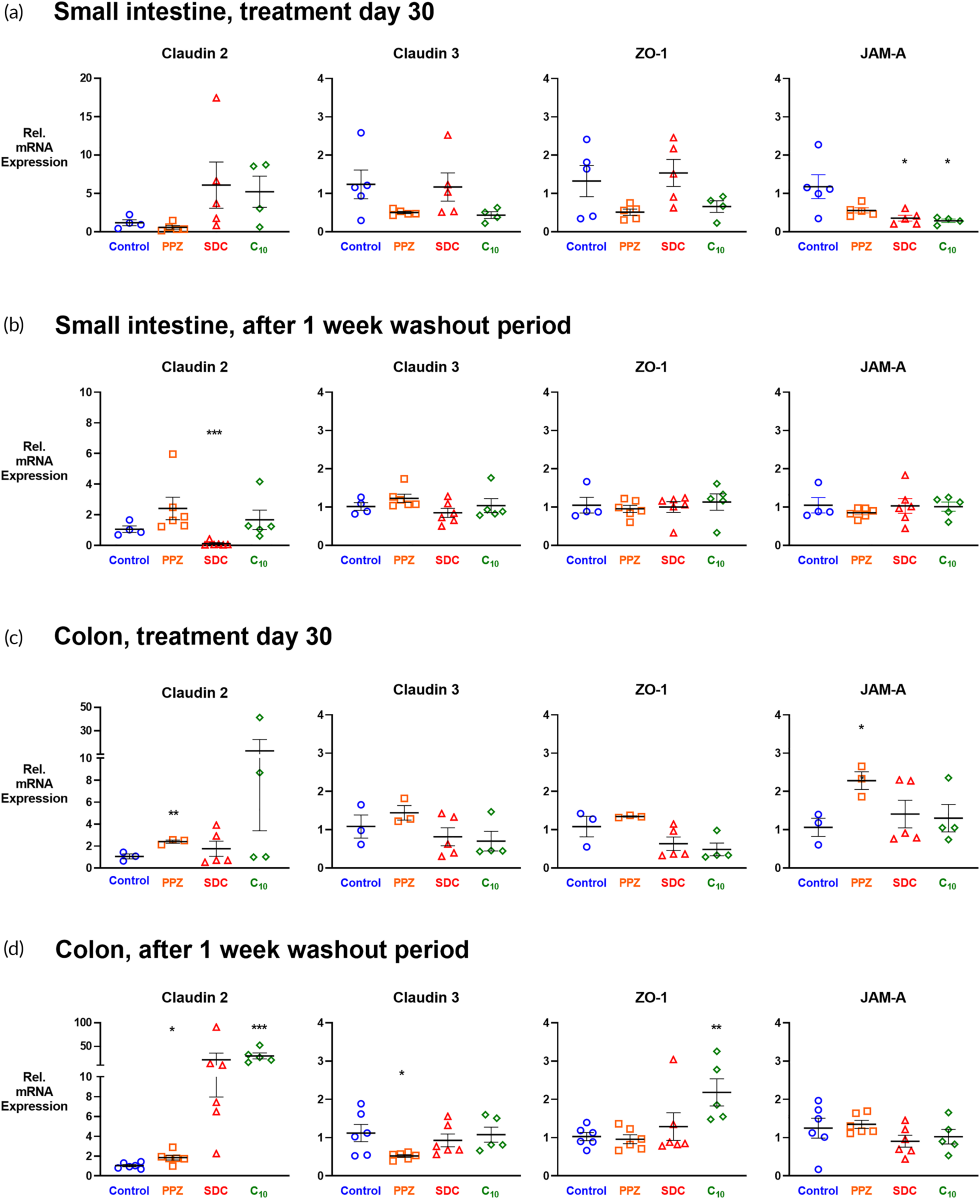
Chronic exposure to permeation enhancers affects gene expression of tight junction proteins. Small intestine and colon tissue samples were collected on treatment day 30 and after a 1‐week washout period, and mRNA expression was determined by qRT‐PCR for four tight junction proteins: pore‐forming *claudin 2*, barrier‐forming *claudin 3*, *ZO‐1*, and *JAM‐A*. (a) On treatment day 30, *JAM‐A* expression in the small intestine decreased for the SDC and C_10_ groups. (b) None of the expression differences persisted after the washout period, but *claudin 2* expression was lower for the SDC group compared to control. (c) In the colon, only PPZ treatment increased expression compared to control. (d) In the colon, after washout, PPZ treatment altered the expression of *claudins 2* and *3*, and C_10_ elevated *ZO‐1* expression. *n* = 6, error bars represent SEM, *p < 0.05, **p < 0.01, ***p < 0.001 by unpaired, two‐tailed Student's t‐test.

Based on the data presented in Figure 2, one dose of permeation enhancer changed the gene expression of several tight junction proteins; therefore, we examined the effects of chronic exposure to these enhancers. Tissue samples were collected from the small intestines and colons of mice sacrificed either on treatment day 30 or after the washout period, and the mRNA expression of *claudin 2*, *claudin 3*, *ZO‐1*, and *JAM‐A* were measured by qRT‐PCR (Figure 5).

On treatment day 30, average *JAM‐A* expression in the small intestine decreased for all enhancer‐treated groups, with only SDC and C_10_ causing statistically significant differences (Figure 5a). However, these decreases in *JAM‐A* expression did not persist after the washout period, indicating that any changes are not permanent (Figure 5b). Interestingly, after the washout period, *claudin 2* expression in the small intestine of SDC‐treated mice decreased compared to the control group. In the colon, only PPZ changed expression compared to control (Figure 5c) with the difference in *JAM‐A* expression resolving after the washout period. For the C_10_ group, expression levels of *claudin 2* and *ZO‐1* were elevated after the washout period (Figure 5d).

### 3.6 Intestinal architecture shows no damage by permeation enhancers as assessed by confocal microscopy

Because of the altered gene expression observed in permeation enhancer‐treated mice, we asked whether these changes affected the architecture of the intestine. To determine this, we used immunofluorescence staining and microscopy to visualize the architecture and tight junction arrangement within samples of small intestine tissue collected from mice sacrificed on treatment day 30. Sections from each treatment group were stained with Hoechst to visualize the nuclei, anti‐claudin 3 antibody with an AlexaFluor 488‐tagged secondary antibody, and anti‐ZO1 tagged with AlexaFluor 594.

**FIGURE 6:**
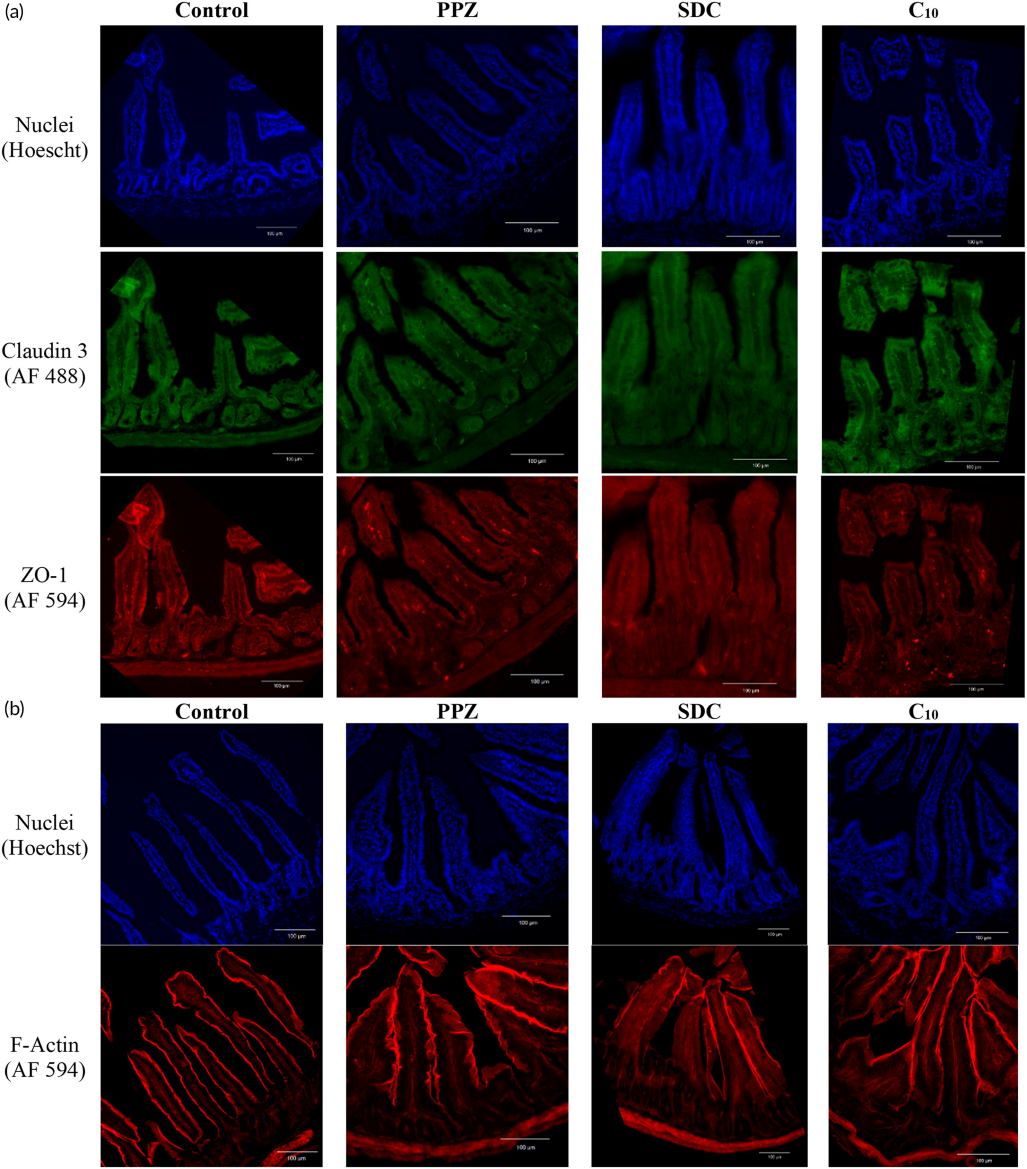
Intestinal architecture and tight junction protein localization were not affected by 4 weeks of treatment with permeation enhancers. (a) Sections of small intestine from mice receiving PBS, PPZ, SDC, or C_10_ were stained for nuclei (blue, Hoechst), the barrier‐forming claudin 3 (green, AF488), and the tight junction protein ZO‐1 (red, AF594). (b) Separate sections were stained for nuclei (blue, Hoechst) and F‐actin (red, AF594).

Representative images are shown in Figure 6a. In all samples, we observed intact villi with no cell sloughing at the tips. Claudin 3 and ZO‐1 were localized at epithelial cell junctions, with clear outlines of the nuclei visible in each sample. Because there were no evident differences in the localization patterns of these proteins between the control and enhancer groups, we concluded that tight junctions did not rearrange in response to treatment. Some samples showed fracturing, which we attribute to the cryosectioning and slide preparation process. In addition, sections from each group were stained with Hoechst to visualize the nuclei and phalloidin tagged with AlexaFluor 594 to visualize F‐actin (Figure 6b). Again, the villi are observed to be intact, with F‐actin surrounding each epithelial cell and the luminal side of each villus. The architectural characteristics of small intestine were preserved in all samples, including intact villus tips and no shortening of the villus‐crypt axis. These types of changes to villi structure have been reported in studies of intestinal damage.^33^, ^34^, ^35^

## 4 DISCUSSION

The clinical translation of oral permeation enhancers has been limited by an incomplete understanding of their mechanisms and impact on long‐term intestinal health. There is consistent and significant skepticism that safe permeation enhancer use is possible.^35^, ^36^, ^37^ The most commonly cited concerns include (1) that increased intestinal permeability will result in the absorption of toxic molecules from the intestinal lumen and (2) that prolonged use will permanently decrease the barrier function of the intestinal epithelium.^35^

We were thus motivated to design a study to address some of these common concerns. Specifically, we characterized the short‐ and long‐term effects of daily oral permeation enhancer administration in mice. We included three permeation enhancers with distinct mechanisms of action. To our knowledge, the paracellular enhancer, 1‐phenylpiperazine (PPZ), and the transcellular enhancer, sodium deoxycholate (SDC), have not been studied for safety or efficacy in vivo.^23^ These enhancers were compared to sodium caprate (C_10_), which is an approved food additive and widelyresearched permeation enhancer that fluidizes the lipid membrane of the epithelial cells and rearranges tight junctions.^15^

One major finding of this study is that the three enhancers caused no permanent increases in intestinal permeability (Figure 3). Previous studies of repeat oral administration of C_10_ in rodent and dog models found similar results as determined by pharmacokinetic profiling.^38^ We noted that SDC and PPZ maintained efficacy each week and did not gradually increase permeability, which would have indicated damage to the epithelium. Studies of PPZ and SDC have been limited to the Caco‐2 cell culture model and ex vivo studies using tissue from rats. However, although these models lack the repair mechanisms of the in vivo intestine, recovery of intestinal barrier integrity was observed after a single dose of the permeation enhancer at effective concentrations.^20^, ^21^, ^22^, ^24^, ^39^

We noted an increase in FD4 serum concentration variability for SDC‐treated mice over the course of treatment, with the same mice having the highest FD4 serum concentrations each week. This observation that individuals respond variably to permeation enhancement is consistent with published studies.^40^ Additionally, daily handling of animals and their associated stress responses may have contributed to variability.

One long‐standing concern in the field of oral delivery is that repeated dosing of permeation enhancers will allow pathogens to cross the intestinal epithelium. This concept of undesired and harmful xenobiotic absorption with the use of permeation enhancers is thoroughly reviewed by McCartney, et al.^35^ Here, we use the marker molecule 4 kDa FITC‐dextran, which is much smaller than lipopolysaccharide, endotoxins, or bacteria (>100 kDa). It is unlikely that the absorption of these large pathogens would be affected by chronic permeation enhancer treatment if, as we found, FD4 absorption did not change with a month of enhancer use.

Further, no absorption of the disease‐associated protein, zonulin, was detected during the month‐long study. Zonulin is an endogenous protein that regulates intestinal permeability by contributing to the disassembly and rearrangement of tight junction proteins. Its pathogenic counterpart, zonula occludens toxin, is produced by Vibrio cholerae and has been studied as a permeation enhancer.^41^, ^42^, ^43^ While there are fewer studies on zonulin, several publications have shown that serum zonulin levels are elevated in humans and rodents with disease‐induced increases in intestinal permeability.^30^, ^31^ We measured the concentration of zonulin in serum samples from mice on treatment day 30 and after the recovery period. None of the enhancer treatments caused statistically significant increases in this protein, suggesting that long‐term treatment did not have an adverse cumulative effect on intestinal barrier function.

PPZ has been shown to affect paracellular permeability in previous work using in vitro and ex vivo models, and the gene expression data collected in this work allows us to extend our mechanistic understanding in vivo.^7^, ^20^, ^21^, ^22^, ^44^ After one dose of enhancer, PPZ significantly decreased the expression of *claudin 3* and *ZO‐1* in the small intestine, and *claudin 3*, *ZO‐1*, and *JAM‐A* in the colon. These differences mirror observations in Caco‐2 monolayers and ex vivo rat tissue that PPZ causes rearrangement of ZO‐1 and actin, a cytoskeletal protein that extensively contacts the tight junctions via ZO‐1.^8^, ^20^, ^22^ After 30 days of treatment, *claudin 3*, *ZO‐1*, and *JAM‐A* expression in the small intestines of PPZ‐treated mice were decreased compared to control mice (*p* = 0.0859, 0.0867, and 0.0875, respectively). None of these changes persisted after the washout period, however, suggesting that the changes are not permanent.

In contrast to PPZ's paracellular mechanism, SDC functions via membrane fluidization of the epithelial cells, which causes increased transcellular transport.^7^, ^23^, ^45^ Aligning with this mechanistic characterization, tight junction gene expression in SDC‐treated mice varied less compared to control mice than it did for PPZ‐ or C_10_‐treated mice. C_10_ is a fatty acid that has effects both on the integrity of the lipid membrane and the tight junctions of the intestinal epithelium, but reassuringly, most of the tight junction expression changes seen for C_10_‐treated mice resolved after the washout period.^46^ Very few studies have quantitatively assessed gene expression changes following permeation enhancer treatment, but one recent study showed that the use of a permeation enhancing polymeric nanoparticle caused downregulation of claudin 4 protein expression after 2 h with corresponding upregulation of *claudin 4* gene expression in the hours after the permeation enhancer treatment was removed.^47^ This may be due to the natural repair mechanisms that exist to regulate tight junction distribution and function.

The largest fold‐change values were found for c*laudin 2* expression in the colons of SDC‐ and C_10_‐treated mice (Figure 5c,d). Increased *claudin 2* expression, particularly in the colon, is seen in studies of inflammatory bowel disease and ulcerative colitis; however, increased *claudin 2* expression in those diseases is always accompanied by other markers of intestinal damage and inflammation.^48^, ^49^, ^50^ In the absence of permeability increases (Figure 3), damage to the mucosa seen in imaging (Figure 6), persistent diarrhea or other fecal abnormalities (Figure 4c), weight loss (Figure 4a), or increased inflammatory markers in the serum (Figure 4b), we are not concerned that this observed upregulation of *claudin 2* indicates the onset of a colitis‐like state. Finally, the tight junction complex is made up of many different types of proteins with interconnected regulation systems. It is unlikely that an expression change for one tight junction protein would majorly compromise the intestinal barrier without corresponding changes to other tight junction proteins.

The results of this study suggest that repeated use of three different permeation enhancers did not alter intestinal health or barrier function in a substantive way. One limitation of this study, however, is that we examined only one concentration for each enhancer. Enhancer behavior and mechanism can vary considerably as a function of concentration^20^, ^22^, ^51^, ^52^; therefore, caution is needed when extending these findings to other permeation enhancer doses. Additionally, this study examined a single dose per day. More research is needed to determine whether these results apply to formulations dosed multiple times a day, insulin being one prominent example.

## 5 CONCLUSION

Intestinal permeation enhancers have long shown promise in enabling of oral protein delivery; however, their use is often hampered by short‐ and long‐term toxicity concerns. Here, we demonstrated that the well‐characterized enhancer, sodium caprate, and the novel permeation enhancers, 1‐phenylpiperazine and sodium deoxycholate, are safe and effective after 1 month of daily use in a mouse model. This study shows the value of including longitudinal in vivo studies in reports of novel permeation enhancer development and encourages additional research on permeation enhancers in oral macromolecular formulations.

## AUTHOR CONTRIBUTIONS

**Katherine C. Fein:** Conceptualization (lead); formal analysis (lead); investigation (lead); methodology (lead); writing – original draft (lead); writing – review and editing (lead). **John Gleeson:** Investigation (supporting); methodology (supporting); writing – review and editing (supporting). **Kyle Cochran:** Investigation (supporting); methodology (supporting); writing – review and editing (supporting). **Nicholas Lamson:** Conceptualization (supporting); investigation (supporting); writing – review and editing (supporting). **Rose Doerfler:** Investigation (supporting); writing – review and editing (supporting). **Jilian R. Melamed:** Investigation (supporting); writing – review and editing (supporting). **Kathryn A. Whitehead:** Conceptualization (supporting); funding acquisition (lead); writing – review and editing (supporting).

## CONFLICT OF INTEREST

The authors have no conflicts of interest to declare.

## Data Availability

The data that support the findings of this study are available from the corresponding author upon reasonable request.
